# Influenza Myopericarditis and Pericarditis: A Literature Review

**DOI:** 10.3390/jcm11144123

**Published:** 2022-07-15

**Authors:** Milan Radovanovic, Marija Petrovic, Michel K. Barsoum, Charles W. Nordstrom, Andrew D. Calvin, Igor Dumic, Dorde Jevtic, Richard D. Hanna

**Affiliations:** 1 Mayo Clinic Alix School of Medicine, Rochester, MN 55905, USA; barsoum.michel@mayo.edu (M.K.B.); nordstrom.cw@mayo.edu (C.W.N.); calvin.andrew@mayo.edu (A.D.C.); dumic.igor@mayo.edu (I.D.); hanna.richard@mayo.edu (R.D.H.); 2 Department of Hospital Medicine, Mayo Clinic Health System, Eau Claire, WI 54703, USA; 3 Icahn School of Medicine at Mount Sinai, New York City, NY 10029, USA; petrovic_marija@yahoo.com (M.P.); djordje965@gmail.com (D.J.); 4 Department of Cardiology, Mayo Clinic Health System, Eau Claire, WI 54703, USA; 5 Department of Internal Medicine, Elmhurst Hospital Center, Elmhurst, NY 11373, USA

**Keywords:** influenza, myopericarditis, myocarditis, pericarditis, cardiac tamponade

## Abstract

Myopericarditis is a rare complication of influenza infection. The presentation may range from mild and frequently unrecognized, to fulminant and potentially complicated by cardiogenic and/or obstructive shock (tamponade), which is associated with high mortality. We performed a review of literature on all influenza pericarditis and myopericarditis cases according to PRISMA guidelines using the PubMed search engine of the Medline database. Seventy-five cases of influenza myopericarditis and isolated pericarditis were identified from 1951 to 2021. Influenza A was reported twice as often as influenza B; however, influenza type did not correlate with outcome. Men and elderly patients were more likely to have isolated pericarditis, while women and younger patients were more likely to have myopericarditis. All included patients had pericardial effusion, while 36% had tamponade. Tamponade was more common in those with isolated pericarditis (41.2%) than myopericarditis (13.8%). Cardiogenic shock was more common in patients with myopericarditis (64%), with an overall mortality rate of 14.7%. Nearly 88% of the recovered patients remained without long-term complications reported. Conclusion: Influenza A appears a more common cause of pericarditis and myopericarditis. Isolated pericarditis was more commonly associated with tamponade but without reported deaths, whereas myopericarditis was more commonly associated with cardiogenic shock and death (19%).

## 1. Introduction

Viral infections have an important impact due to global pandemics and the high burden on societies worldwide [1]. The World Health Organization (WHO) and Center for Disease Control (CDC) estimate 3 to 5 million severe influenza cases around the world with close to 650,000 deaths annually [2]. Although influenza usually causes mild respiratory disease, severe extra-pulmonary complications, such as myocarditis and pericarditis, have been sporadically reported since the 1918 Spanish influenza pandemic [3]. Cardiac complications have been encountered through all major pandemics since then, including Asian influenza in 1957, Hong Kong influenza in 1968, Russian influenza in 1977, and most recently the 2009 swine influenza pandemic [4].

Viral myocarditis remains one of the major causes of acute and chronic (dilated) cardiomyopathy, with the enteroviruses (particularly coxsackievirus B), adenoviruses, parvoviruses, and some herpes viruses being the most common causative pathogens [5]. Due to shared etiology (i.e., cardiotropic viruses), viral myocarditis is commonly accompanied by some degree of pericarditis and vice versa. Therefore, the terms “myopericarditis” or “perimyocarditis” are often used interchangeably in clinical practice. Recent papers and guidelines have attempted to further clarify terminology based on the predominant type of cardiac involvement [6,7,8,9]. In this review, all cases determined to represent a combination of myocarditis and pericarditis will be referred to as myopericarditis—recognizing that this may represent a spectrum of myocarditis-predominant cases with coexistent pericardial involvement to pericarditis-predominant cases with mildly elevated biomarkers. Cases published before the availability of highly sensitive cardiac biomarkers likely could not detect mild coexistent myocarditis; nonetheless, numerous published cases were clearly pericarditis predominant and were categorized as “pericarditis only” if that was the conclusion of the authors. Previous studies report a clinical diagnosis of influenza myocarditis in the 5–10% range, whereas evidence of myocardial involvement can be found in up to 40% of autopsy cases [10,11,12,13]. Influenza pericarditis is considered rare and can occur during the acute infection but also in a post-vial syndrome as a delayed, subacute, and often unrecognized (subclinical) complication due to mild clinical presentation [11].

There are numerous reports and reviews of influenza myocarditis [1,13,14,15,16], however, the literature on myopericarditis or isolated pericarditis complicating influenza infection is limited. Therefore, this study aims to describe and summarize the clinical characteristics, diagnostic and therapeutic approaches, complications, and outcomes of patients suffering from influenza myopericarditis or isolated pericarditis in the published literature.

## 2. Materials and Methods

We performed a literature review of all influenza pericarditis and myopericarditis cases according to preferred reporting items for systematic reviews and meta-analyses (PRISMA) guidelines using the PubMed search engine of the Medline (National Library of Medicine, Bethesda, MD, USA) database, from database inception until 3 January 2022 (no PROSPERO registration number was required for a literature review). A total of 588 original articles were identified that mention the following search keywords (combination of MeSH and non-MeSH terms): “influenza AND pericarditis” OR “influenza AND myopericarditis” OR “influenza AND perimyocarditis” OR “influenza AND tamponade” OR “influenza AND myocarditis”, excluding “*Hemophilus influenzae*”. We excluded cases where the diagnosis was uncertain, either by influenza not being diagnosed or lack of evidence of acute pericarditis/myopericarditis. The diagnosis of acute pericardial involvement was established by identifying at least two of the following diagnostic criteria: pericardial (positional; pleuritic) chest pain, pericardial friction rub, electrocardiographic (ECG) findings of ST-segment elevation and/or PR-segment depression, or pericardial effusion seen on cardiac imaging. Myocardial involvement was defined by a case with elevated cardiac enzymes, new ventricular dysfunction, or tissue abnormalities by endomyocardial biopsy (EMB) and/or cardiac magnetic resonance imaging (CMR). Duplicate articles, articles in languages other than English, abstracts without comprehensive case descriptions, and narrative reviews were all excluded.

**Figure 1:**
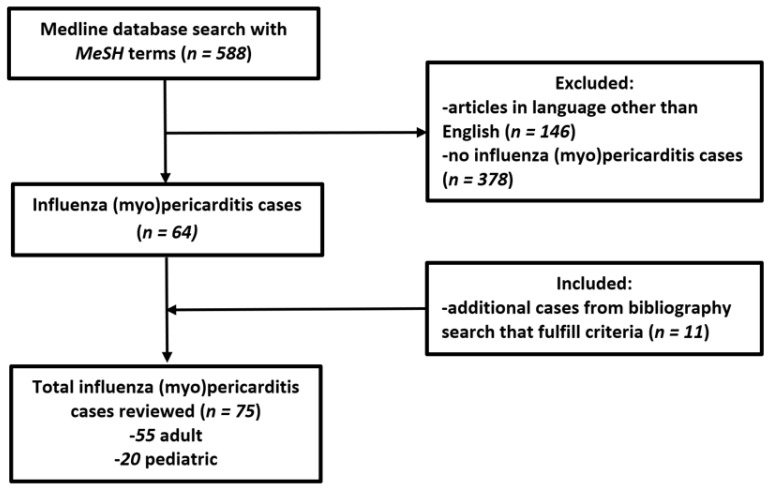
Flow chart of methodology and literature selection according to the PRISMA guidelines.

Two authors (M.R. and D.J.) independently and blindly identified and selected titles, abstracts, and full texts in the database search. Discrepancies in the selected articles were resolved by the third author (I.D.). Additionally, the reference list of selected articles and recent influenza myocarditis review articles [1,14,15,17,18] were searched to identify any additional cases for inclusion in accordance with previously established selection criteria (i.e., cases that had pericardial involvement in addition to myocarditis). The flow chart of detailed article selection and the final cases included in the analysis is illustrated in Figure 1.

We extracted demographic data, co-morbid conditions, presenting symptoms, physical exam findings, laboratory studies, imaging findings (including ECG, echocardiography, and other chest imaging), treatment options, complications, and outcomes.

Data were analyzed by descriptive statistics and expressed as mean ± standard deviation for continuous data, or as frequency and percentages for categorical data. Student t-test and Chi-square tests were used to test the differences between patients in relation to outcome (survival). Univariate regression analysis was used to determine factors associated with mortality. Statistical significance was reported using a *p*-value < 0.05. SPSS statistical software (version 21.0) was used for statistical analysis.

## 3. Results

### 3.1. Demographics

Our literature review identified 75 unique cases (55 adult and 20 pediatric patients) from 56 case reports, and 5 case series describing 2 to 8 patients each, published from 1959 through 2021 [2,12,18,19,20,21,22,23,24,25,26,27,28,29,30,31,32,33,34,35,36,37,38,39,40,41,42,43,44,45,46,47,48,49,50,51,52,53,54,55,56,57,58,59,60,61,62,63,64,65,66,67,68,69,70,71,72,73,74,75,76]. The age of patients in this review ranged from 4 months to 75 years (mean 32.3 ± 18.8 years). Both genders were almost equally represented in the adult population, however, there was a significant predominance of the female sex in the pediatric population (Table 1).

There was no statistical difference in age, gender, or presence of comorbidities in relation to survival (*p* > 0.05).

### 3.2. Presentation

Patients most commonly presented with an acute febrile illness (*n* = 71, 94.7%), followed by tachycardia (*n* = 62, 82.7%), hypotension/shock (*n* = 54, 72%), chest pain (*n* = 36, 48%), and dyspnea (*n* = 34, 45.3%). Findings highly suggestive of pericardial involvement were present in less than half of the patients. These included pericardial friction rub (*n* = 15, 20%), muffled heart sounds (*n* = 8, 10.7%), and pulsus paradoxus (*n* = 5, 6.7%). Whereas pericardial friction rub was more frequently recognized in isolated pericarditis cases (*p* < 0.001), tachycardia and hypotension were more often identified in myopericarditis cases (*p* < 0.001).

At the time of presentation, tamponade was more often recognized in isolated pericarditis cases (7 out of 14, 41.2%; *p* = 0.015), while cardiogenic shock with or without cardiac tamponade was diagnosed only in patients with myopericarditis (37 out of 58, 63.8%; *p* < 0.001). Isolated cardiac tamponade was also encountered in 8 out of 58 myopericarditis cases (13.8%), however, no cardiogenic shock was reported in isolated pericarditis cases. The total number of patients diagnosed with shock was 52 (70% of all reviewed cases) (Table 2).

### 3.3. Evaluation

**Figure 2:**
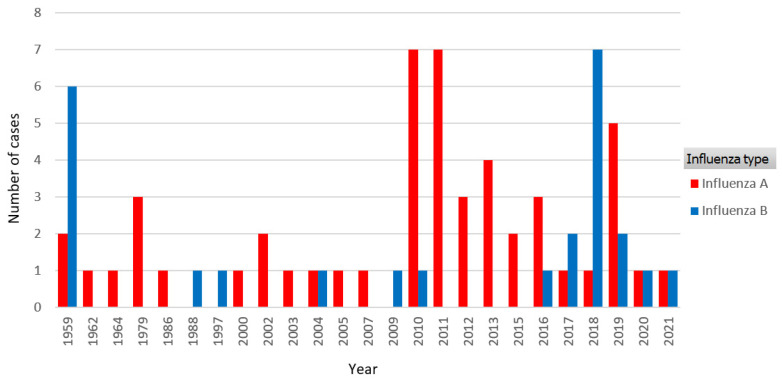
Influenza type distribution over years, with a notably higher incidence of reported cases during and after the 2009 influenza A (H1N1) pandemic.

**Figure 3:**
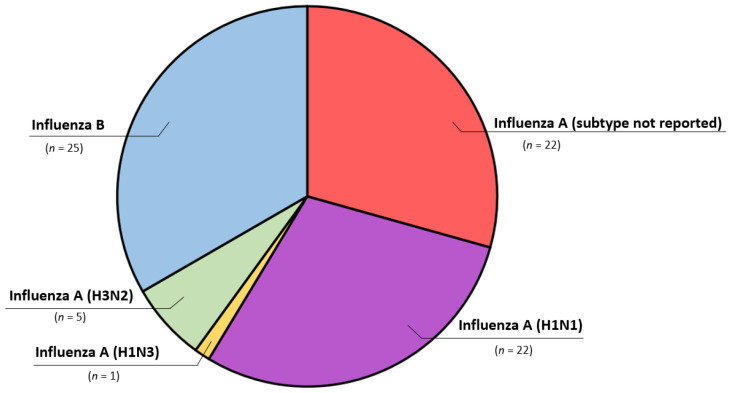
Influenza type and subtype distribution.

All patients tested positive for acute influenza infection, either by nasopharyngeal swab (*n* = 50, 66.7%) or by serology testing (*n* = 25, 33.3%), with the notable predominance of serology testing used in older publications (before the year 2000). Influenza type (A to B, 2:1) did not correlate with worse outcome (*p* > 0.05), however, 9 out of 11 deceased patients had been diagnosed with influenza A. There was a notably higher incidence of reported cases during and after the 2009 influenza A (H1N1) pandemic (30% of all cases) (Figure 2). Other notable subtypes in our review were H3N2 identified in five cases and H1N3 in one case (Figure 3). Elevated cardiac enzymes (either troponin or in older cases creatine-kinase isoenzyme MB) were reported in 44 cases (58.7%) without the relation to survival (*p* > 0.05).

Greater than 80% of cases had reported abnormal ECG, with sinus tachycardia being the most common ECG finding (80%), followed by ST-segment elevation with or without PR-segment depression (*n* = 26, 42.6%), low voltage QRS complexes (*n* = 22, 36.1%), and electrical alternans (*n* = 2, 3.3%). Pericardial effusion was reported in all reviewed cases (100%), although detailed TTE reports were only found in 62 cases (82.6%). TTE findings of decreased left ventricular (LV) or bi-ventricular function or diffuse hypokinesis were found in 42 cases (67.7% of cases with reported TTE), while cardiac tamponade was reported in 27 cases (36%).

Additional diagnostic imaging performed included: computed tomography (CT) of the chest in 13 patients (17.3%), cardiac catheterization in 9 (12%), transesophageal echocardiography (TEE) in 4 (5.3%), and cardiac magnetic resonance imaging (CMR) in 3 patients (4%) [29,46,51]. Endomyocardial biopsy (EMB) was performed in only three patients (4%), while two patients (2.7%) had myocardial tissue samples obtained during the course of mechanical circulatory support (MCS) placement [18,34,65,68,77]. Histopathology was reported in 91% (*n* = 10) of autopsies. Histopathology findings ranged from minimal inflammatory, predominantly lymphocytic infiltrate with edema to myocyte necrosis due to complement-mediated cellular injury (Table 3).

Pericardial fluid influenza reverse transcriptase-polymerase chain reaction (RT-PCR) testing was positive in only four cases [35,43,51,56], while the case from 1962 had influenza virus isolated in chicken kidney cultures [75]. Two patients had identified influenza in the post-mortem heart biopsy, either by viral genome by RT-PCR [54], or by viral particles on electron microscopy [18]. The presence of influenza in the myocardial tissue or pericardial space did not correlate statistically with a worse prognosis (*p* > 0.05), although two patients required subsequent pericardiectomy due to recurrent pericardial effusion [43] and constrictive pericarditis [51].

### 3.4. Treatment and Interventions

Antiviral medications were used in half of the patients. The most commonly used antivirals were neuraminidase inhibitors (NAI), such as oseltamivir (*n* = 33, 44%), peramivir (*n* = 6, 8%), or zanamivir (*n* = 3, 5%), with four cases receiving a combination of two NAI. Amantadine was used in only one case of influenza B myopericarditis, although authors doubted its effectiveness [70].

Anti-inflammatory medications were rarely used and consisted of non-steroidal anti-inflammatory medications (NSAIDs; 17.3%), corticosteroids (16%), and colchicine (10.7%). Intravenous immunoglobulin (IVIG) was used in eight cases (10.7%).

Circulatory support consisted of inotropes/vasopressors (used in 58.7% of the cases) and MCS, including extracorporeal membrane oxygenation (ECMO), in 18 cases (24%), intra-aortic balloon pump (IABP) in 9 (12%), and ventricular assist device (VAD) in 7 cases (9.3%). VAD devices used were Impella in four cases [20,25,34,60], Bi-VAD in two, and catheter-based VAD in one case [18,50,68]. Only 1 out of 17 patients with isolated pericarditis required inotropic/vasopressor support compared to 44 out of 58 myopericarditis cases (*p* < 0.001). Among myopericarditis cases, 41.4% (*n* = 24) required MCS (*p* < 0.001).

Pericardial decompression and drainage were performed in almost half of the cases. Needle pericardiocentesis was predominant (*n* = 28, 37.7%), followed by pericardiectomy (*n* = 4, 5.3%), and pericardial window (*n* = 3, 4%). Two patients required pericardiectomy on subsequent hospitalizations due to recurrent pericardial effusion [43] or pericardial constriction [51].

### 3.5. Complications and Outcome

The most commonly reported complication was a cardiogenic shock (70%), followed by reaccumulation of pericardial effusion (28.6% of cases that had pericardial drainage).

Only 8 out of 64 recovered patients (12.5%) had long-term complications reported. Chronic constrictive pericarditis was reported in three cases [51,73], while recurrent pericardial effusion was seen in two cases [43,61]. Dilatated cardiomyopathy was reported in only 2 patients, one of whom had a history of influenza B myocarditis 16 years prior [33,46].

Non-cardiac complications were pleural effusions in 16 cases (21.3%) and rhabdomyolysis/myositis in 13 cases (17.3%). Additionally, some form of pneumonia and respiratory distress was present and reported in most cases.

Most of the patients had a positive outcome and recovered from the infection (84%), while one patient required a heart transplant [18]. The mortality rate was 14.7% (*n* = 11 patients), occurring within 2 days from presentation due to cardiogenic shock and/or tamponade, illustrating the fulminant nature of the illness (Table 4). There was a patient reported by Takehana et al. that expired after 24 days [66]. The patient had a biphasic course, initially recovering from influenza myopericarditis after 2 weeks, followed by decompensation that was hypothesized by authors to be due to either viral re-infection or a post-infectious autoimmune process.

Mortality was equally observed in the adult and pediatric populations, and only in myopericarditis cases (*p* = 0.05). There was no statistical difference in age, sex, influenza type/subtype, or vasopressor support in relation to survival (*p* > 0.05). The univariate regression model suggested lower mortality in myopericarditis patients when MCS was used (*p* < 0.05, R^2^ = 0.210, X2 = 30.268) (Table 5).

## 4. Discussion

### 4.1. Presentation and Diagnostics

Cardiovascular complications during influenza infection are often under-recognized, particularly in young and previously healthy patients due to their non-specific and often mild severity [11]. However, worsening dyspnea, chest pain, and hemodynamic instability usually point toward cardiac involvement [11]. Cardiac symptoms typically occur quite rapidly, usually between 4 and 9 days after the onset of initial influenza symptoms [11,77]. Furthermore, fulminant myopericarditis has a rapid onset and usually occurs within 2 weeks from the initial presentation. After the first 2 weeks of myopericarditis, patients either recover with complete functional and histologic resolution or expire due to refractory cardiogenic shock and/or tamponade [78].

All reviewed cases had illness development and presentation to the healthcare facility within 2 weeks. Several patients presented with syncope, with one case (a healthy 11-year-old girl) reportedly experiencing syncope leading to death at home [69]. Tachycardia was a frequent but non-specific sign of myocardial involvement, but some patients had a dramatic presentation with shock. Expedited echocardiographic imaging was often helpful to characterize the type of the shock, rapidly distinguishing cardiogenic from an obstructive shock (i.e., tamponade) [79]. Additionally, echocardiography was helpful to evaluate for the presence of pericardial effusion, LV systolic dysfunction, and even aneurysm formation [9,80]. In our review, pericardial effusion was a universal finding (100%), while generalized hypokinesis and LV systolic dysfunction were reported in 67.7%.

EMB remains the gold standard for diagnosis of myocarditis, however, in clinical practice, it is performed primarily to identify cases of giant-cell or eosinophilic myocarditis as these entities may respond to immunosuppressive therapy. As such, EMB is generally discouraged by most societies if the clinical diagnosis appears certain [81]. Placement of MCS may offer an opportunity to concurrently obtain tissue for microscopic analysis [13]. When performed, histopathology is usually paucicellular with minimal inflammatory, predominantly lymphocytic infiltrate, interstitial edema, and cardiomyocyte necrosis. Influenza is rarely detected from myocardial tissue or pericardial fluid [2,82]. Animal models have demonstrated that the severity of the myocardial injury does not correlate with viral titers in the heart and that viral concentrations are much higher in the respiratory tract compared to the myocardium [83].

In the cases reported to date, CMR was infrequently utilized, but emerging literature suggests that CMR may be helpful to identify cases of active myocarditis [5,84] and for long-term follow-up [22]. Additionally, CMR may be useful in localizing sites for EMB [5] to facilitate the PCR identification of the viral genome or for providing information regarding the presence of giant cells (worse prognosis) versus a lymphocytic-rich infiltrate (better prognosis) [85]. In this series, only three patients had CMR, and they demonstrated findings consistent with acute myocarditis and pericardial thickening with late enhancement [29,46,51].

### 4.2. Treatment and Prevention

Exacerbation of existing cardiovascular disease, including heart failure [86] and coronary artery disease [87], is the most commonly cited mechanism by which influenza leads to cardiovascular morbidity and mortality [88], and is a major reason why vaccination is recommended for patients with preexisting cardiovascular diseases [89]. Whether vaccination can prevent pericarditis and myopericarditis is less clear [11,86].

Supportive management is the backbone of severe influenza treatment, but antiviral medications are widely used for both inpatient and outpatient influenza management [90]. Although previous studies of NAI showed that early administration reduces the symptom duration and hospital stay [91,92], some studies show mortality benefits in critically ill patients if initiated later [93]. Whereas oseltamivir is utilized in both inpatient and outpatient settings, peramivir and zanamivir are used only for hospitalized patients [90]. There are individual case reports of successful influenza myocarditis treatment with peramivir [60,94] and zanamivir [28,95]. Peramivir was often used in combination with either oseltamivir [46,50] or zanamivir [41], and in two cases with systemic corticosteroids [53] and intravenous immunoglobulin (IVIG) with variable outcomes [60]. The effectiveness of the next-generation medications, such as baloxavir-marboxil (selective cap-dependent endonuclease inhibitor), has not been reported.

Patients unresponsive to the maximum supportive therapy may benefit from immunomodulatory treatment given the significant role of the inflammation and cytotoxin-storm in the pathogenesis of severe influenza infections [5]. The benefit of immunomodulatory therapy (such as IVIG) in the management of fulminant myocarditis has been studied; and while some studies showed a potential benefit [96] others did not [97]. In our review, IVIG was used only in fulminant myocarditis cases, and always in combination with one of the antiviral medications.

The European Society of Cardiology (ESC) guidelines recommend the use of anti-inflammatory medications, such as NSAIDs and colchicine, for acute pericarditis [9]. Corticosteroids are suggested as a second-line therapy for patients who do not respond or are intolerant to NSAIDs and colchicine [9]. Although corticosteroids may provide rapid symptom control, there is concern that steroid therapy (particularly at higher doses) may predispose to recurrent pericarditis [98]. In a comprehensive review from 2008 by Imazio et al., low to moderate corticosteroid dosages (e.g., prednisone 0.2–0.5 mg/kg per day) were associated with a lower recurrence rate compared to high dosages (1.0 mg/kg per day) [98,99]. A meta-analysis from 2010 by Lotrionte et al. reported a similar finding: low-dosage corticosteroids were associated with lower recurrence rates compared to high-dosage corticosteroids [100]. In acute influenza myopericarditis, the benefit of systemic corticosteroids is controversial, and early avoidance has been suggested to minimize negative effects in the early phases of viral replication [1].

Fulminant myopericarditis cases with cardiogenic shock may require inotropic support along with short-term MCS (e.g., IABP, ECMO, or VAD) as a bridge to myocardial recovery [11]. Almost half of the analyzed cases were managed with a similar approach: initially with inotropic infusion, but ultimately two-thirds progressed to temporary MCS, including simultaneous ECMO with IMPELLA (ECMELLA) in three cases [20,25,60].

### 4.3. Complications and Outcomes

With fulminant influenza myocarditis, patients often have a prolonged and complicated hospital course with significant mortality [101]. Management with circulatory support may be necessary [11]. Patients who survive fulminant myocarditis tend to recover within a few weeks to months, with an excellent long-term outcome and an estimated 10-year survival rate above 93% [45,102]. Mortality in this review was found to be 14.7%, significantly lower than the mortality in recent reviews of influenza myocarditis that ranged from 24% [17] to 35% [32], attributed mainly to fulminant myocarditis and sudden cardiac death [11]. This is probably due to the inclusion in our review of patients with isolated pericarditis in whom no deaths were observed. However, even amongst our cases with myopericarditis, the mortality rate was 19%, which may be due to the inclusion of pericarditis-predominant cases with a better prognosis.

Chronic dilated cardiomyopathy or chronic constrictive pericarditis were rarely observed despite severe clinical manifestations and fulminant course, and complete functional recovery was common (87.5%) [11]. It is worth mentioning that one healthy teenage patient required a heart transplant after an H1N1 influenza myopericarditis with development of severe dilated cardiomyopathy [18]. In rare cases, restrictive cardiomyopathy or recurrent pericardial effusion may occur as long-term sequelae; this occurred in five cases in this review [43,51,61,73,74]. Two of these cases had influenza A virus genome identified in the pericardial sample, and both cases required pericardiectomy [43,51]. Furthermore, one patient had recurrent pericardial effusions, but also had systemic lupus erythematosus (SLE), a condition that by itself is associated with recurrent pericardial effusions [61]. A case series of three end-stage renal disease (ESRD) patients receiving hemodialysis reported acute influenza A pericarditis, which was complicated by chronic pericarditis in two cases [73]. Authors hypothesized that ESRD and chronic uremia predisposed patients to chronic pericarditis. One case reported LV inferior pseudo-aneurysm with akinetic inferolateral wall following an influenza A myopericarditis diagnosis, however, this patent had an inferolateral myocardial infarction 6 years prior [67]. The authors concluded that the pseudoaneurysm was a consequence of influenza myopericarditis, as his earlier echocardiographic examinations were unremarkable.

Influenza infections also predispose patients to a secondary bacterial infection, most commonly pneumonia. The pericardium may also be affected, with rare case reports of *Methicillin-resistant Staphylococcus Aureus* (MRSA), *Group A Streptococcus*, and *Streptococcus pneumoniae* pericarditis [103,104,105,106]. This occurs due to multiple immunological mechanisms, including damage of the tracheobronchial epithelial layer and local immunologic response suppression (i.e., bacterial clearance), causing easier bacterial adherence and translocation [107]. Secondary bacterial infections following influenza significantly complicate clinical course and cause increased morbidity and mortality [107].

## 5. Conclusions

Influenza is most often a self-limited respiratory illness, but severe cardiac complications, such as pericarditis leading to tamponade and fulminant myocarditis leading to shock, may occur. Cardiac involvement should be considered in patients of any age with chest pain, tachycardia, and hemodynamic instability within 2–4 weeks of symptom onset. Our study summarized more than 60 years of patient data from case reports and case series, showing that influenza A appears more commonly associated with cardiac involvement than influenza B. No deaths were reported in cases with isolated pericarditis, although they were more commonly associated with cardiac tamponade. Myopericarditis cases were more commonly associated with cardiogenic shock, requiring MCS in 41.4% of cases, and had a fatality rate of 19%.

## 6. Limitations of the Study

Limitations of our study are inherent to the nature of this type of literature review and include selection bias, as well as publication bias leading to predominant fulminant cases publication. An additional limitation of our literature review is that we have included only cases in the English language and ones that were published in journals that are indexed in the PubMed/MEDLINE database. Although these strict criteria were implemented to avoid low-quality case reports, we recognize that we might have missed some high-quality cases if they did not meet our pre-selection criteria.

## Figures and Tables

**Table 1:** The epidemiology, demographics, clinical presentation, diagnostic findings, and outcome of influenza myopericarditis cases.

**Demographic Characteristics**	**n**	**M to F Ratio**	**Age Range (Years)**	**Mean Age (Years)**
Adult	55 (73.3%)	28:27	18–75	40.5 ± 14.8
Pediatric	20 (26.7%)	3:17	0.25–17	9.7 ± 4.9
Total	75 (100%)	31:44	0.25–75	32.3 ± 18.8
**Co-morbidities**				
Adult				
Not present	26 (47.3%)			
Present	29 (52.7%)			
Hypertension and alcohol use	each in 4 (7.3%)			
CAD (previous MI), advanced CKD/ESRD, asthma, tobacco dependence	each in 3 (5.4%)			
Obesity and hyperlipidemia	each in 2 (3.6%)			
Previous Influenza B myocarditis (16 years prior), DMT2, hypothyroidism, primary biliary cirrhosis, diverticulitis, breast cancer, SLE, MS, TBI, Down syndrome, schizophrenia, marijuana and cocaine use, previous infection with TB, syphilis, gonorrhea	each in 1 (3.6%)			
Pediatric				
Not present	17 (85%)			
Present	3 (15%)			
Viral myocarditis	1 (5%)			
Asthma	1 (5%)			
Rheumatic fever	1 (5%)			
**Clinical presentation**		**Symptom duration**	**Range (days)**	**Mean (days)**
Febrile (“flu-like”) illness	71 (94.7%)	Reported–66 (88%)	1–42	6.9 ± 6.4
Tachycardia	62 (82.7%)	Not reported–9 (12%)		
Hypotension/Shock	54 (72%)			
Chest pain	36 (48%)			
Dyspnea	34 (45.3%)			
Pericardial friction rub	15 (20%)			
Elevated JVP	10 (13.3%)			
Abdominal pain	8 (10.7%)			
Muffled heart sound	8 (10.7%)			
Nausea/vomiting	8 (10.7%)			
Collapse/syncope	6 (8%)			
Pulsus paradoxus	5 (6.7%)			
Altered mental status/lethargy	5 (6.7%)			
**ECG findings**				
Normal or not reported	14 (18.7%)			
Abnormal				
ST elevation and/or PR depression	26 (42.6%)			
Low voltage QRS complexes	22 (36.1%)			
Electrical alternans	2 (3.3%)			
**Echocardiography findings**				
Performed	62 (82.7%)			
Decreased EF or diffuse hypokinesis	42 (67.7%)			
Pericardial effusion				
Without tamponade physiology	38 (61.3%)			
With tamponade physiology	24 (38.7%)			
Not reported, although authors reported pericardial effusion in all 13 cases with tamponade in 3 cases	13 (17.3%)			
**Treatment**				
Antivirals (Oseltamivir/Peramivir/Zanamivir)	33 (44%)/6 (8%)/3 (5%)			
NSAIDs	13 (17.3%)			
Corticosteroids	12 (16%)			
Colchicine	8 (10.7%)			
IVIG	8 (10.7%)			
Circulatory support				
Inotropes/vasopressors	44 (58.7%)			
Mechanical				
ECMO	18 (24%)			
Intra-aortic balloon pump	9 (12%)			
Ventricular assist device	7 (9.3%)			
Pericardiocentesis	28 (37.3%)			
Pericardiectomy/Pericardial window	4 (5.3%)/3 (4%)			
**Outcome and complications**				
Recovered	63 (84%)			
Long-term complications				
No		55 (87.3%)		
Yes		8 (12.7%)		
Chronic (constrictive) pericarditis		3 (4.8%)		
Recurrent pericardial effusions		2 (3.2%)		
Mild LV dysfunction		2 (3.2%)		
LV pseudoaneurysm		1 (1.6%)		
Awaiting transplant	1 (1.3%)			
Deceased	11 (14.7%)			

Legend: CKD—chronic kidney disease; ESRD—end-stage renal disease; CAD—coronary artery disease; MI—myocardial infarction; DMT2—diabetes mellitus type 2; SLE—systemic lupus erythematosus; MS—multiple sclerosis; TBI—traumatic brain injury; TB—tuberculosis; JVP—jugular venous pressure; EF—ejection fraction; NSAIDs—non-steroidal anti-inflammatory drugs; IVIG—intravenous immunoglobulin; ECMO—extracorporeal membrane oxygenation; LV—left ventricular.

**Table 2:** Characteristics and comparison of myopericarditis vs isolated pericarditis cases.

	Myopericarditis	vs.	Pericarditis
**Number of cases**	58 (77.3%)		17 (22.7%)
**Shock type**	Cardiogenic (*n* = 25, 43.1%)		Obstructive (tamponade) (*n* = 7, 41.2%)
	Combined (*n* = 12, 20.7%)		
	Obstructive (tamponade) (*n* = 8, 13.8%)		
**Gender predominance**	Female		Male (***p* = 0.008**)
**Age**	Younger patients (30 ± 19 years)		Older patients (39 ± 19 years; *p* = 0.091)
**Co-morbidities**	No impact (*p* > 0.05)		No impact (*p* > 0.05)
**Initial physical exam**	Tachycardia and hypotension (shock) (***p* < 0.001**)		Pericardial friction rub (***p* < 0.001**)
**Circulatory support (CS)**	44 (75.7%)		1 (5.9%)
**Mechanical CS**	24 (41.4%)		None
**Deceased**	11 (*p* = 0.05)		None

**Table 3:** Histopathologic findings of myocardial tissue.

Reference	Age/Sex	Influenza Type	Sampling	Histopathology
**Jiménez-Méndez et al. (2019)** [26]	35 M	Influenza A	EMB	Minimal inflammatory infiltrate, CD3-positive cells
**Lefeuvre et al. (2018)** [2]	14 F	Influenza A (H3N2)	Autopsy	Myocardial necrosis with contraction bands and interstitial edema with an abundant mononuclear inflammatory infiltrate
**Roto et al. (2018)** [31]	57 F	Influenza B	Autopsy	Myocardial necrosis with infiltration of CD3-positive lymphocytes
**Siskin et al. (2017)** [34]	22 F	Influenza B	EMB	Myocardial necrosis through complement-mediated cellular injury without evidence of interstitial infiltrates
**Davidovic et al (2016)** [39]	19 M	Influenza A (H1N1)	Autopsy	Extensive zones of necrosis with degenerative cardiomyocytes and inflammatory neutrophilic and lymphocytic infiltrate
**Lee et al. (2012)** [48]	8 F	Influenza A (H1N1)	Autopsy	Widespread contraction band myofiber necrosis with increased interstitial cellularity (mostly CD68-positive monocytes and CD8-positive T lymphocytes and no eosinophils)
**Kumar et al. (2011)** [18]	17 F	Influenza A (H1N1)	Tissue sampling at the time of VAD placement	Extensive myocyte necrosis with the confirmation of viral particles by electron microscopy
**Khouzam et al (2011)** [50]	36 M	Influenza A (H1N1)	Autopsy	Myocardial interstitium exhibited edema and an inflammatory infiltrate, rich in lymphocytes and macrophages
**Frank et al. (2010)** [54]	5 F	Influenza B	Autopsy	Moderate interstitial infiltration of lymphocytes, as well as neutrophils and eosinophils, were found. Influenza B RNA was detected in cardiac tissue
**Puzelli et al. (2010)** [56].	11 F	Influenza A (H1N1)	Autopsy	Mild inflammation, modest infiltration of histiocytes (CD68-positive), and myocellular necrosis
**Gerberding et al. (2004)** [64]	18 M	Influenza A	Autopsy	Cardiac myocyte hypertrophy and a patchy lymphohistiocytic infiltrate in perivascular areas associated with interstitial edema. Focal contraction-band myocyte necrosis and scattered intravascular fibrin thrombi
**Tabbutt et al (2004)** [65]	4 F	Influenza B	EMB	Mildly congested myocardium, with interstitial fibrosis and rare lymphocytes. Electron microscopy showed mildly pleomorphic mitochondria and the absence of viral inclusions
**Takehana et al. (2003)** [66]	75 M	Influenza A	Autopsy	Marked inflammatory cell infiltration, mainly composed of mononuclear cells, with myocardial degeneration and necrosis, and interstitial edema
**McGovern et al. (2002)** [68]	30 F	Influenza A	Tissue sampling at the time of VAD placement	Focal interstitial fibrosis, diffuse lymphocytic infiltrate
**Nolte et al. (2000)** [69]	11 F	Influenza A (H3N2)	Autopsy	Transmural, sparse, patchy infiltrates of lymphocytes and neutrophils associated with myocyte necrosis and nuclear debris

Legend: EMB—Endomyocardial biopsy; VAD—Ventricular assist device.

**Table 4:** Fatal influenza myopericarditis cases.

Reference	Age/Sex	Co-morbidities	Presenting Symptoms	Symptom Duration	Influenza Type	Cardiac Involvement	Cardiogenic Shock	Cardiac Tamponade	Pericardial Drainage	Medical Management	Circulatory Support	Time to Death
**Thomas et al. (2019)** [27]	23 F	None	AFI, dyspnea,	3 days	Influenza A (H1N1)	Myopericarditis	Yes	No	No	Oseltamivir	Vasopressors/inotropes	1 day
**Lefeuvre et al. (2018)** [2]	14 F	None	AFI, dyspnea, collapse	not reported	Influenza A (H3N2)	Myopericarditis	Yes	Yes	No	None	Vasopressors/inotropes	1 day
**Roto et al. (2018)** [31]	57 F	None	AFI, dyspnea, AMS	7 days	Influenza B	Myopericarditis	No	Yes	Pericardiocentesis (90 mL)	Not reported	Vasopressors/inotropes	not reported
**Davidovic et al (2016)** [39]	19 M	None	AFI, chest pain	2 days	Influenza A (H1N1)	Myopericarditis	Yes	No	No	NSAIDs	Vasopressors/inotropes	several hours
**Lee et al. (2012)** [48]	8 F	Previous viral myocarditis	AFI, chest pain, vomiting	2 days	Influenza A (H1N1)	Myopericarditis	Yes	No	No	Oseltamivir, IVIG	Vasopressors/inotropes	2 days
**Khouzam et al (2011)** [50]	36 M	childhood asthma	AFI, dyspnea, nausea, diarrhea	21 days	Influenza A (H1N1)	Myopericarditis	Yes	No	Pericardiocentesis (700 mL)	Oseltamivir, peramivir	vasopressors/inotropes, catheter based VAD	18 h
**Frank et al. (2010)** [54]	5 F	None	AFI, abdominal pain	7 days	Influenza B	Myopericarditis	Yes	No	No	Not reported	Vasopressors/inotropes	1 day
**Puzelli et al. (2010)** [56]	11 F	None	AFI, dyspnea	3 days	Influenza A (H1N1)	Myopericarditis	No	Yes	Autopsy (150 mL)	None	No	not reported
**Gerberding et al. (2004)** [64]	18 M	Obesity, HLD	AFI, pleuritic chest pain, mottled skin	5 days	Influenza A	Myopericarditis	Yes	No	Autopsy (400 mL)	Not reported	Vasopressors/inotropes	36 h
**Takehana et al. (2003)** [66]	75 M	not reported	AFI	not reported	Influenza A	Myopericarditis	Yes	No	No	Not reported	IABP	24 days
**Nolte et al. (2000)** [69]	11 F	None	AFI, collapse	7 days	Influenza A (H3N2)	Myopericarditis	No	Yes	Autopsy (40 mL)	None	No	Died before hospitalization

Legend: HLD—hyperlipidemia; AFI—acute febrile illness; AMS—altered mental status; NSAIDs—non-steroidal anti-inflammatory drugs; IVIG—intravenous immunoglobulin; VAD—ventricular assist device; IABP—intra-aortic balloon pump.

**Table 5:** Regression analysis in a prediction of patients’ outcomes.

	Univariate Regression Analysis		
Variable	*p* Value	OR	95% CI for OR
Sex	0.564	1.806	0.243–13.444
Age	0.931	1.002	0.949–1.059
Influenza type/subtype	0.940	0.931	0.147–5.889
Tamponade (recognized on presentation)	0.570	0.570	0.082–3.965
Cardiogenic shock	0.157	4.983	0.538–46.180
Mechanical circulatory support	**0.047**	0.094	0.009–0.971
Vasopressor support	0.863	0.755	0.031–18.375
